# Triple synchronous malignant tumors of colon, appendix and liver: A case report with literature review

**DOI:** 10.12669/pjms.291.2277

**Published:** 2013

**Authors:** Shen Guoliang, Huang Dongsheng

**Affiliations:** 1 Dr. Shen Guoliang, Zhejiang Provincial People’s Hospital, Shangtang Road Number 168, Hangzhou, Zhejiang Province, China.; 2 Dr. Huang Dongsheng, Zhejiang Provincial People’s Hospital, Shangtang Road Number 168, Hangzhou, Zhejiang Province, China.

**Keywords:** Synchronous Malignancy

## Abstract

Synchronous cancers are defined as malignant tumors that occur simultaneously. Each tumor must be primary which eliminate the possibility of being metastatic lesion of the other. If three separate organs are involved, that is so-called triple synchronous malignancy with very low morbidity. We report a case of a 33 year old male patient with triple synchronous malignancies at the colon, appendix and liver.

## Case Reports

**Fig.1:**
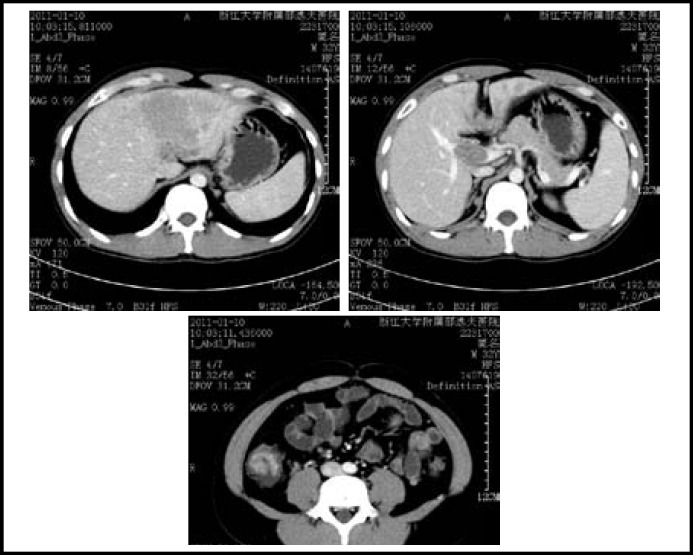
: A left liver mass (10cmx8cm) with portal vein cancerous embolus and a right colon mass (3cmx3cm) with incomplete colon obstruction

 A 33-year-old man was referred to our hospital with upper abdominal pain and poor appetite for two months; he claimed weight loss of 10 Kg. Medical history revealed no particular past history or familial history except chronic virus hepatitis B infection for 10 years. The fecal occult blood test was positive and serum AFP level was elevated (382280ng/ml). Enhanced abdomen CT scan demostrated a mass of 10cmx8cm in the left lobe of liver with portal vein cancerous embolus and a mass in the right colon with partial incomplete obstruction (Fig.1). Colonoscopy showed a mass in the right colon (65cm to anus). Pathological result confirmed it was adenocarcinoma.

 A right colectomy was performed. The liver mass was unresectable due to infiltration to the porta hepatis. Postoperative pathological result showed highly-differentiated adenocarcinoma of right colon, partial myxoadenocarcinoma, carcinoid of appendix and hepatocellular carcinoma of left liver. Recovery after operation was uneventful and the patient was discharged 10 days after surgery. The patient underwent two times of TACE after operation and died of liver failure 8 months later since his diagnoses.

## Discussion

 Triple synchronous malignancies are relatively rare and it accounts for about 1.8%-3.9% of all primary tumors reported in the literature.^1^^,^^2^ According to the Warren’s criteria, such tumours occurring at different locations must be histologically malignant and separated by normal mucosa. Each tumour must not be a metastasis of another.^3^ Meortal proposed that two primary tumors found within 6 months could be defined as synchronous, otherwise (more than 6 months) is metachronous. In our case, the three tumors were synchronous with different sites of liver, colon and appendix confirmed by postoperative pathological exam. 

**Table-I:** Surgical statistics of synchronous and metachronous primary triple tumors

	*In one system*	*In different systems*	*Cases of surgery*
Synchronous (19 cases)	13	6	12/19
Metachronous (16 cases)	4	12	12/16

*35 cases in total in the Table-I, 2 of 37 cases were excluded due to non availability of clinical details

 Literature review of past 20 years showed that there were 37 cases of triple primary tumors which have been reported so far. Those cases were mainly elderly males (94.1%),^4^^-^^8^ with an average age of 70.1 years, Among the 37 cases reported, 19 cases were synchronous (51.4%), and there was no particular history except that one patient had atomic bomb exposure^9^, one had bone marrow transplantation and two had silicosis diseases.^10^^,^^11^ Even though they were triple primary tumors, most cases had non-specific symptoms such as weight loss and anorexia. More than 90% of the triple primary tumors were diagnosed by CT or endoscopy^4^^,^^5^^,^^12^ with one discovered by PET-CT.^13^ Three cases of tumor specimens underwent cancer genetic testing including p53, p16, p21 and cyclinD1, which, however no significant difference were dectected.^6^^,^^12^

 The definition of synchronous and metachronous tumors is different in literature. The synchronous tumors were located in the same system (68.42%, 3/19), while the metachronous tumors in different systems (75%, 12/16). Synchronous triple primary tumors has a lower resectability than metachronous (63.16% to 75%) tumors^14^^-^^16^, as synchronous tumors may need a lager range removal of the lesions (Table-I). Only 11 of the reported 37 cases had described the prognosis. The longest survival time of the synchronous patients were 13 months, who was still alive till reported;^7^ the longest survival time of the metachronous patients were 18 years.^17^

 The diagnosis and treatment for synchronous triple primary tumors were still challenging. We suggest for elderly male patient of more than 70 years, a comprehensive assessment should be performed if a primary tumor of one system was diagnosed in order to avoid missed diagnosis of synchronous triple primary tumors.
